# Corrigendum: Engineering and optimising deaminase fusions for genome editing

**DOI:** 10.1038/ncomms16169

**Published:** 2017-10-09

**Authors:** Luhan Yang, Adrian W. Briggs, Wei Leong Chew, Prashant Mali, Marc Guell, John Aach, Daniel Bryan Goodman, David Cox, Yinan Kan, Emal Lesha, Venkataramanan Soundararajan, Feng Zhang, George Church

Nature Communications 7: Article number: 13330 ; DOI: 10.1038/ncomms13330 (2016); Published 11 02 2016; Updated 10 09 2017.

The original version of this Article contained an error in the email address of the corresponding author George Church. The correct email is gchurch@genetics.med.harvard.edu. The error has been corrected in the HTML and PDF versions of the Article.

