# Supplementary material for: Engineering and optimising deaminase fusions for genome editing
Source: Nat Commun. 2016 Nov 2;7:13330. doi: 10.1038/ncomms13330 (PMC5097136; doi:10.1038/ncomms13330)
Supplement: Supplementary Information — Supplementary Figures 1-7, Supplementary Tables 1-2 and Supplementary Notes 1-16. [file ncomms13330-s1.pdf]

Supplementary Figure 1

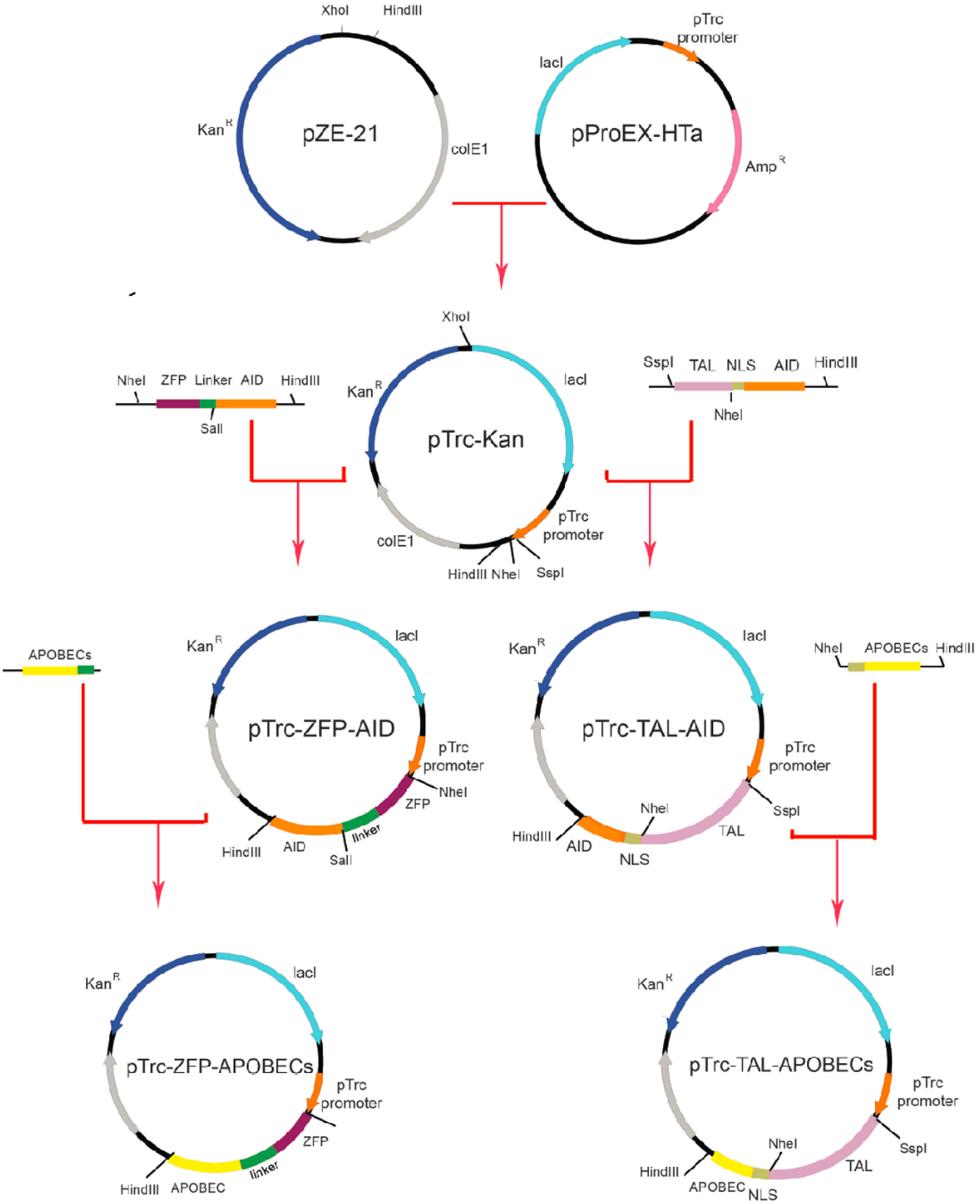

Supplementary Figure 1 | Schematic representation of the design and construction of targeted deaminase expression vectors. The expression vector pTrc-Kan was derived from pZE-21 and pROEx-HTa. We inserted ZF-AID fusions into pTrc-Kan to generate pTrc-ZF-AIDs. From pTrc-ZF-AIDs, we swapped AID with other APOBECs to further construct pTrc-ZF-APOBECs. In parallel, pTrc-TALE-AIDs were constructed by inserting TAL-AID fusions into pTrc-Kan. pTrc-TALE-APOBECs were generated by replacing the AID coding sequence with APOBECs coding sequences.

Supplementary Figure 2

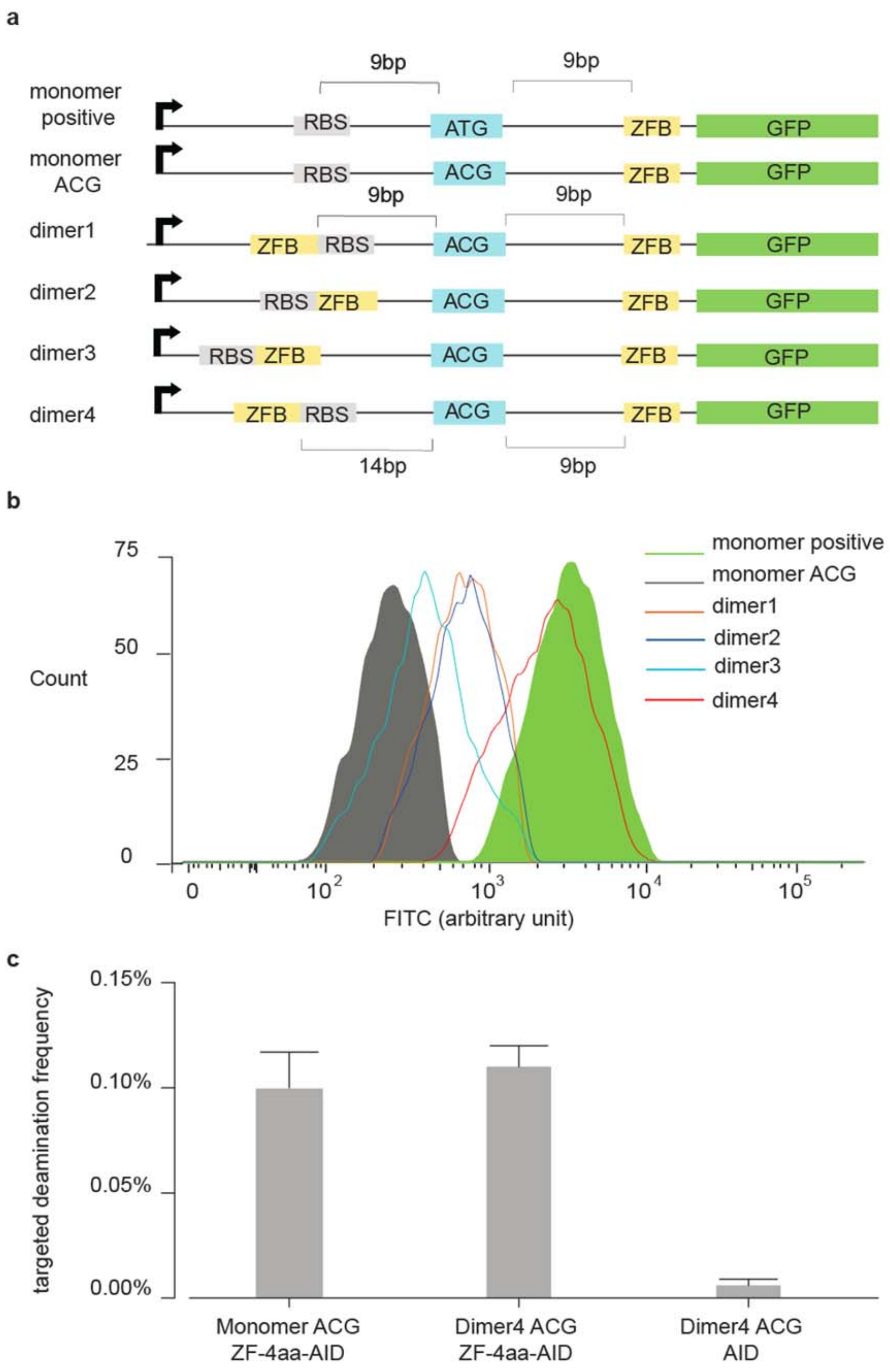

Supplementary Figure 2 | Test of targeted deaminase frequency on the reporter with two ZF binding sites. **a,** Schematic representation of the modified GFP reporters with two ZF binding sites. In the monomer reporter, a ZF binding site (ZFB) lies 9bp downstream of the start codon (in blue). In the dimer reporter constructs, an additional ZFB lies either 9bp (dimer1 and dimer3), 6bp (dimer2), or 14bp (dimer4) upstream of the start codon. Arrows indicate promoter, RBS indicate position of ribosome binding site. **b,** Overlap histogram of GFP expression level from the different reporters. Dimer1, 2 and 3 exhibited significant overlaps with the negative control (uninduced monomer ACG reporter), suggesting that the alterations to the length or sequence between the RBS and start codon compromised the translation of GFP. In contrast, the dimer4 reporter showed distinct GFP fluorescence, so we chose it for the following test. **c,** Targeted deamination frequency on dimer and monomer reporters. ZF-4aa-AID expression led to similar GFP rescue frequency in both the dimer4 ACG and monomer ACG GFP reporter systems. Conversely, AID expression alone did not result in any detectable GFP rescue signal, indicating that the ZF-4aa-ZFP monomer was able to specifically target the genomic site. Targeted deamination frequency was quantified via percentage of GFP-expressing cells in the population. All error bars indicate s.d.. Experiments were performed in triplicate.

Supplementary Figure 3

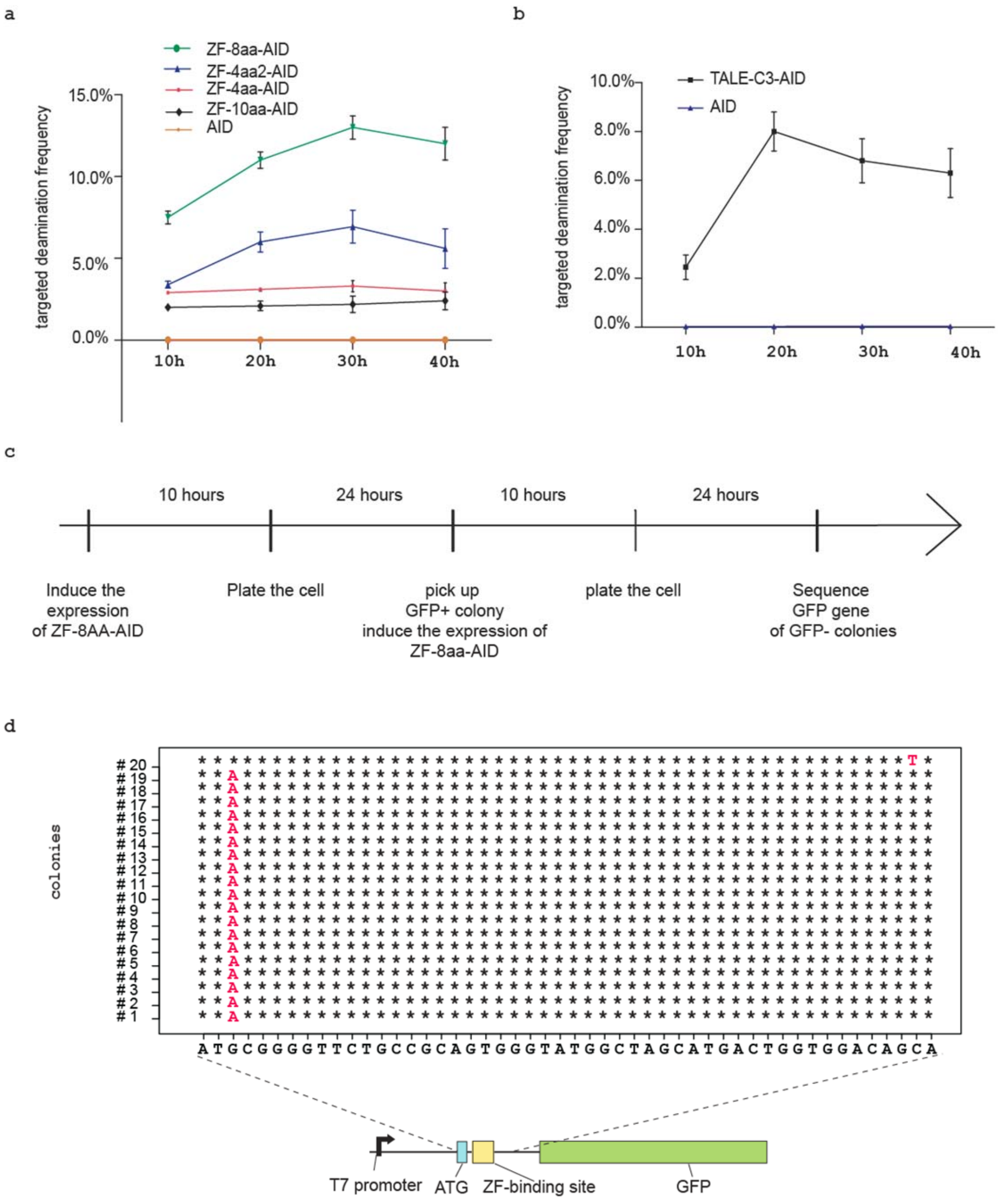

Supplementary Figure 3 | Secondary mutations led to the decline of GFP rescue efficiency **a,** Targeted deamination frequency peaked following 30 hours of ZF-AID induction and dropped after that. The targeted deaminase frequencies were measured by flow cytometry analysis of GFP expression. Bacterial culture was diluted 1:100 every 10 h to maintain continuous cell proliferation. **b,** Targeted deamination frequency as measured by GFP+ cell fraction peaked following 20 hours of TALE-AID induction and dropped after that. Bacterial culture was diluted 1:100 every 10hrs to maintain continuous cell proliferation. **c,** Time line depicting the experiment design to capture secondary mutations. **d,** Sanger DNA sequencing revealed that prolonged ZF-AID induction led to secondary mutations that abolished the expression of GFP. 1kb of the *gfp* gene was sequenced over 20 GFP- colonies; only the mutated part is shown in the table. The original sequence is listed below and the schematic graph of the GFP cassette shows the corresponding positions of this sequence. "*" indicates positions where the sequence is identical with the wild type *gfp*. Red letters indicate the mutated bases. All error bars indicate s.d.. Experiments were performed in triplicate.

Supplementary Figure 4

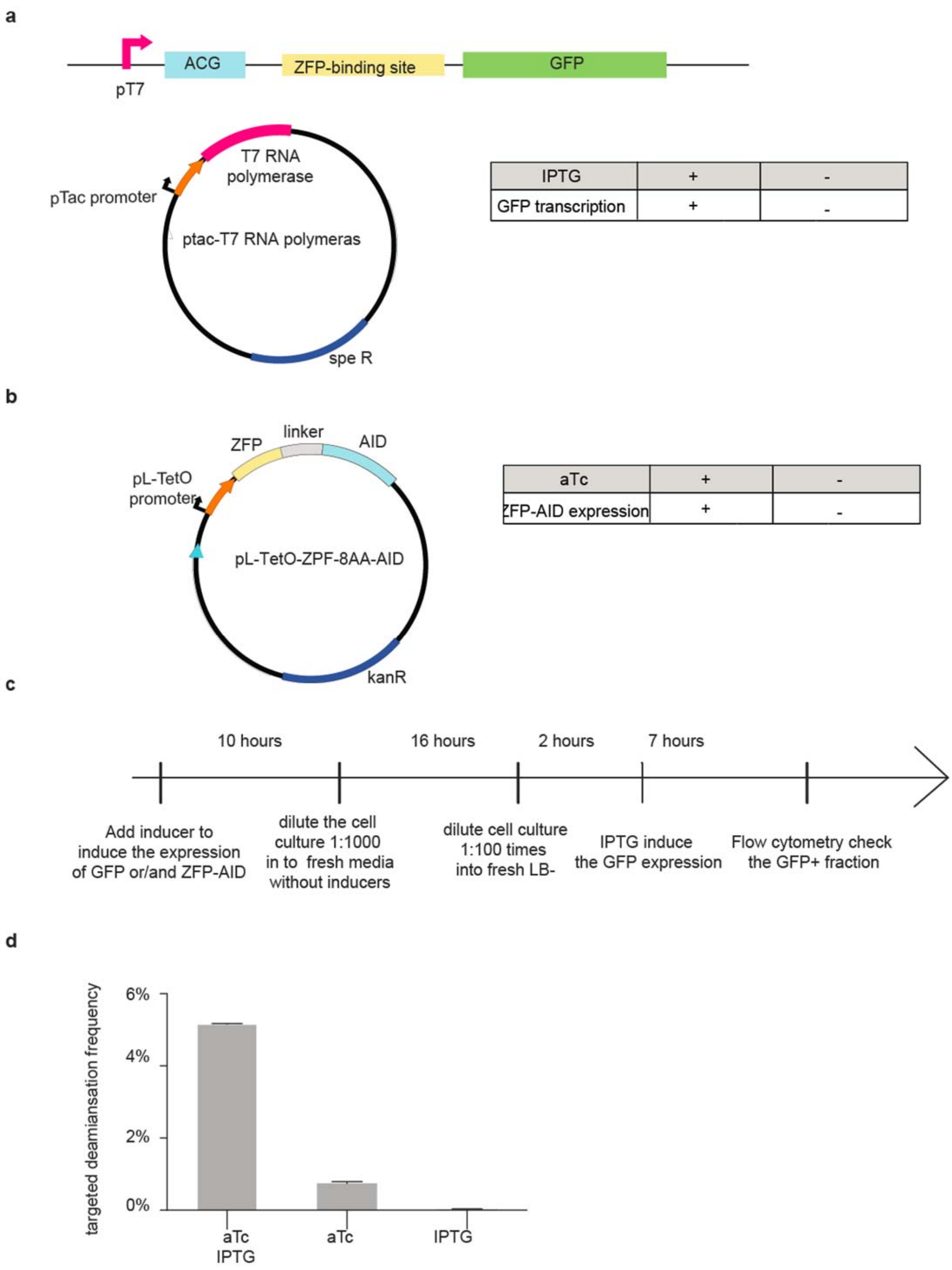

Supplementary Figure 4 | Active transcription enhances targeted deamination**. a,** Schematic representation of the transcription control of the GFP reporter. This GFP was transcribed by T7 RNA polymerase which is transcribed by an IPTG inducible promoter pTac. **b,** Schematic representation of the transcription control of ZF-AID. ZF-AID was transcribed from the pL-TetO promoter which was modulated by the TetR protein (constitutively expressed) and the inducer aTc. **c,** Time line depicting the experiment design. **d,** Targeted deamination frequency with/without GFP transcription**.** The bacterial culture was induced with IPTG, aTc and IPTG&aTC for 10 hours, and then diluted 1000-fold into fresh media without any inducer overnight. Cell culture was diluted again 100-fold into fresh media with IPTG to check for the expression of GFP. Targeted deamination frequency was quantified via percentage of GFP-positive cells in the population.

Supplementary Figure 5

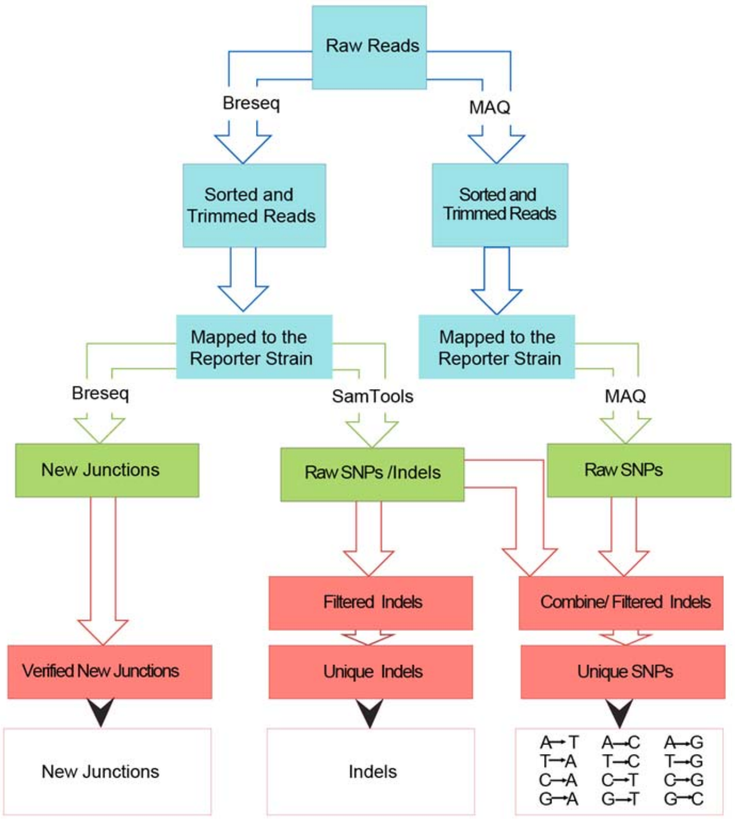

Supplementary Figure 5 | Flow map of the whole-genome sequence data analysis. Breseq and MAQ were used independently to assign the raw reads to different strains and align the reads to the reference genomes. After alignment, we used Samtools and MAQ to identify single nucleotide substitutions (SNSs), Breseq to identify new genomic junctions and Samtools to call indels.

Supplementary Figure 6

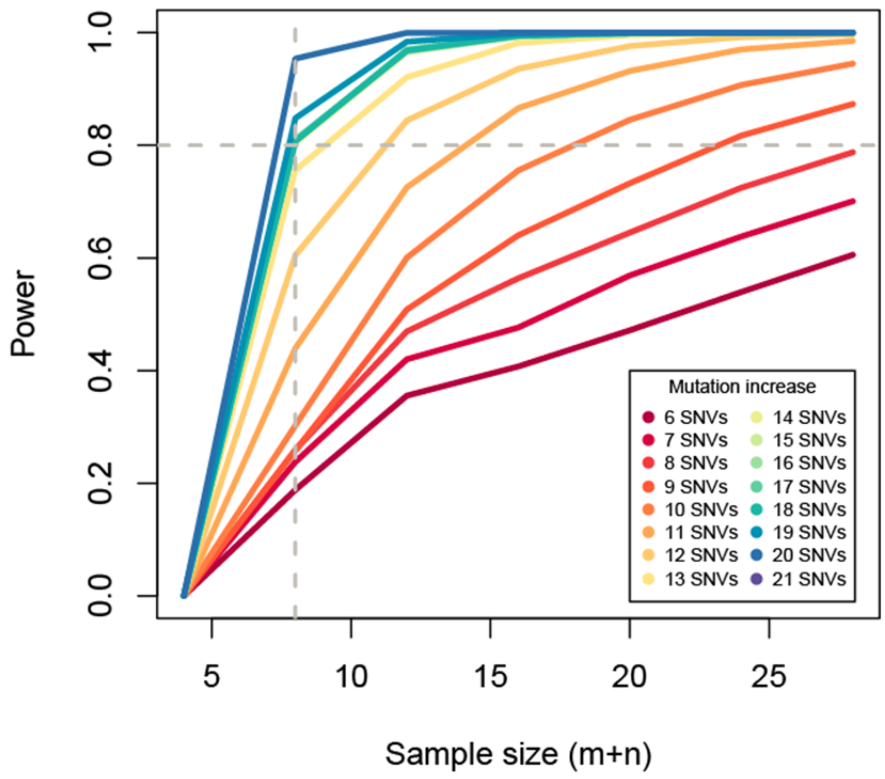

Supplementary Figure 6 | Sensitivity simulations for the Wilcoxon test of numbers of genome SNV comparison

Supplementary Figure 7

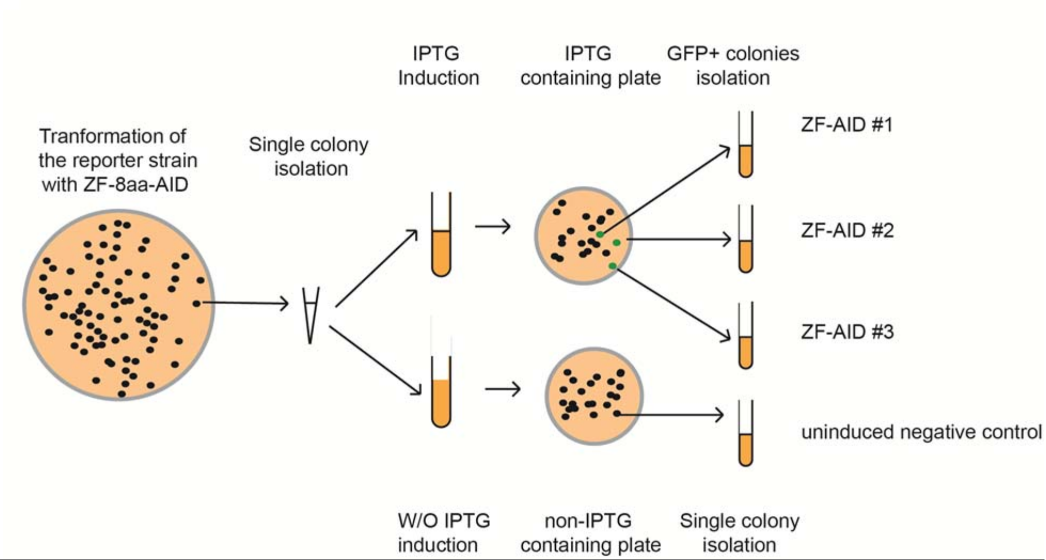

Supplementary Figure 7 | Illustration of the whole-genome sequence library preparation.

Supplementary Table 1

Summary of the Next Generation Sequence Analysis of the *E.coli* strains induced with ZF-8aa-AID

Strain	negative		ZF-AID #1		ZF-AID #2		ZF-AID #3	
	Breseq	MAQ	Breseq	MAQ	Breseq	MAQ	Breseq	MAQ
Sorted Reads	5297284	5168108	3409951	3504126	1886216	1933461	4373651	4256740
Mapped to EcNR2	5164973	4093566	3212263	3183401	1774630	1357434	4266413	2952749
% mapped to the genome	97.5%	79.2%	94.2%	91%	94.1%	70.2%	97.5%	69.4%
Average coverage (X)	92.7		55.3		30.9		73.8	
Total SNVs	100		110		101		97	
Total single nucleotide substitutions	88		102		95		91	
Total Indels	12		8		6		6	
Unique SNVs	31		41		32		28	
Unique subsitutions	22		36		29		25	
Unique indels	9		5		3		3	
A→T	0		0		0		0	
A→C	0		0		0		0	
A→G	1		2		3		4	
C→A	0		0		0		0	
C→T	7		18		10		12	
C→G	0		0		0		1	
G→A	11		15		15		7	
G→T	0		0		0		0	
G→C	0		0		0		0	
T→A								
T→C	3		1		1		1	
T→G								
C→T/G→A mutations in the WRC motif	9		25		15		15	
C→T/G→A mutations in the non-WRC motif	9		13		9		14	
Other type of substitutions	4		3		4		6	

Supplementary Table 2

Summary of the Next Generation Sequence Analysis of the *E.coli* strains induced with TALE-C3-AID

Strain	negative		TALE-AID #1		TALE-AID #2		TALE-AID #3	
	Breseq	MAQ	Breseq	MAQ	Breseq	MAQ	Breseq	MAQ
Sorted Reads	879775	1319162	3269195	4143893	3934097	3890959	2671258	2650878
Mapped to EcNR2	752217	633922	3218793	2425436	3720863	3574572	2605760	2522975
% mapped to the genome	85.5%	48.1%	98.5%	58.5%	94.6%	91.9%	97.5%	95.2%
Average coverage (X)	12.9		55.5		68.7		48.2	
Total SNVs	106		119		106		118	
Total single nucleotide substitutions	99		110		99		111	
Total Indels	7		9		7		7	
Unique SNVs	21		34		21		33	
Unique subsitutions	18		29		18		30	
Unique indels	3		5		3		3	
A→T	0		0		0		0	
A→C	0		0		1		0	
A→G	1		0		3		0	
C→A	0		0		0		0	
C→T	5		15		4		12	
C→G	0		0		0		0	
G→A	7		11		7		14	
G→T	0		0		1		0	
G→C	0		0		0		0	
T→A	0		0		0		0	
T→C	5		3		2		4	
T→G	0		0		0		0	
C→T/G→A mutations in the WRC motif	9		13		9		14	
C→T/G→A mutations in the non-WRC motif	3		13		2		12	
Other type of substitutions	6		3		7		4	

Supplementary Note 1

ZF -AID constructs and the PCR primer sequences

ZF coding sequence is capitalized, linker sequence is highlighted in green, AID coding sequence is un-capitalized. NheI cutting site is labeled in Red, HindIII cutting site is labeled in Blue.

ZF -4aa-AID:

GCTAGCCCCAGAGTGAGAACCGGTTCTAAGACACCTCCCCACGAGAGGCCTTTTCAGTGTAGAATTTGTATGCGTAATTTTTC
TAGGTCCGATGTGCTGGCCAATCACACAAGGACTCACACTGGTGAAAAGCCCTTCCAATGTAGAATTTGTATGCGCAATTTTT
CTCAATCTTCTACTCTGACTAGACATCTGAGGACCCACACAGGCGAAAAGCCTTTCCAGTGCAGAATTTGTATGAGAAATTTT
TCTGAAAGACAGGGTCTGAAAAGACATCTGAAGACACATACAGGTGAAAAAGGATCCGGTGGTGGTTCTgacagcctcttgat
gaaccggaggaagtttctttaccaattcaaaaatgtccgctgggctaagggtcggcgtgagacctacctgtgctacgtagtga
agaggcgtgacagtgctacatccttttcactggactttggttatcttcgcaataagaacggctgccacgtggaattgctcttc
ctccgctacatctcggactgggacctagaccctggccgctgctaccgcgtcacctggttcacctcctggagcccctgctacga
ctgtgcccgacatgtggccgactttctgcgagggaaccccaacctcagtctgaggatcttcaccgcgcgcctctacttctgtg
aggaccgcaaggctgagcccgaggggctgcggcggctgcaccgcgccggggtgcaaatagccatcatgaccttcaaagattat
ttttactgctggaatacttttgtagaaaaccacgaaagaactttcaaagcctgggaagggctgcatgaaaattcagttcgtct
ctccagacagcttcggcgcatccttttgcccctgtatgaggttgatgacttacgagacgcatttcgtactttgggactt

ZF -4aa2-AID:

GCTAGCCCCAGAGTGAGAACCGGTTCTAAGACACCTCCCCACGAGAGGCCTTTTCAGTGTAGAATTTGTATGCGTAATTTTTC
TAGGTCCGATGTGCTGGCCAATCACACAAGGACTCACACTGGTGAAAAGCCCTTCCAATGTAGAATTTGTATGCGCAATTTTT
CTCAATCTTCTACTCTGACTAGACATCTGAGGACCCACACAGGCGAAAAGCCTTTCCAGTGCAGAATTTGTATGAGAAATTTT
TCTGAAAGACAGGGTCTGAAAAGACATCTGAAGACACATACAGGTGAAAAAGGATCCCTGCGTGGTTCTgacagcctcttgat
gaaccggaggaagtttctttaccaattcaaaaatgtccgctgggctaagggtcggcgtgagacctacctgtgctacgtagtga
agaggcgtgacagtgctacatccttttcactggactttggttatcttcgcaataagaacggctgccacgtggaattgctcttc
ctccgctacatctcggactgggacctagaccctggccgctgctaccgcgtcacctggttcacctcctggagcccctgctacga
ctgtgcccgacatgtggccgactttctgcgagggaaccccaacctcagtctgaggatcttcaccgcgcgcctctacttctgtg
aggaccgcaaggctgagcccgaggggctgcggcggctgcaccgcgccggggtgcaaatagccatcatgaccttcaaagattat
ttttactgctggaatacttttgtagaaaaccacgaaagaactttcaaagcctgggaagggctgcatgaaaattcagttcgtct
ctccagacagcttcggcgcatccttttgcccctgtatgaggttgatgacttacgagacgcatttcgtactttgggactt

ZF-8AA-AID

GCTAGCCCCAGAGTGAGAACCGGTTCTAAGACACCTCCCCACGAGAGGCCTTTTCAGTGTAGAATTTGTATGCGTAATTTTTC
TAGGTCCGATGTGCTGGCCAATCACACAAGGACTCACACTGGTGAAAAGCCCTTCCAATGTAGAATTTGTATGCGCAATTTTT
CTCAATCTTCTACTCTGACTAGACATCTGAGGACCCACACAGGCGAAAAGCCTTTCCAGTGCAGAATTTGTATGAGAAATTTT
TCTGAAAGACAGGGTCTGAAAAGACATCTGAAGACACATACAGGTGAAAAAGGATCCTCTGGTGGTGGTCTGGGGTCGACTga
cagcctcttgatgaaccggaggaagtttctttaccaattcaaaaatgtccgctgggctaagggtcggcgtgagacctacctgt
gctacgtagtgaagaggcgtgacagtgctacatccttttcactggactttggttatcttcgcaataagaacggctgccacgtg
gaattgctcttcctccgctacatctcggactgggacctagaccctggccgctgctaccgcgtcacctggttcacctcctggag
cccctgctacgactgtgcccgacatgtggccgactttctgcgagggaaccccaacctcagtctgaggatcttcaccgcgcgcc
tctacttctgtgaggaccgcaaggctgagcccgaggggctgcggcggctgcaccgcgccggggtgcaaatagccatcatgacc
ttcaaagattatttttactgctggaatacttttgtagaaaaccacgaaagaactttcaaagcctgggaagggctgcatgaaaa
ttcagttcgtctctccagacagcttcggcgcatccttttgcccctgtatgaggttgatgacttacgagacgcatttcgtactt
tgggactt

ZF-11AA-AID:

GGTTCTGGTGGTGGTGGTTCTGGTGGTGGTGGTGCTAGCCCCAGAGTGAGAACCGGTTCTAAGACACCTCCCCACGAGAGGCC
TTTTCAGTGTAGAATTTGTATGCGTAATTTTTCTAGGTCCGATGTGCTGGCCAATCACACAAGGACTCACACTGGTGAAAAGC
CCTTCCAATGTAGAATTTGTATGCGCAATTTTTCTCAATCTTCTACTCTGACTAGACATCTGAGGACCCACACAGGCGAAAAG
CCTTTCCAGTGCAGAATTTGTATGAGAAATTTTTCTGAAAGACAGGGTCTGAAAAGACATCTGAAGACACATACAGGTGAAAA
AGGATCCGGTTCTGGTGGTGGTGGTTCTGGTGGTGGTGGTgacagcctcttgatgaaccggaggaagtttctttaccaattca
aaaatgtccgctgggctaagggtcggcgtgagacctacctgtgctacgtagtgaagaggcgtgacagtgctacatccttttca

ctggactttggttatcttcgcaataagaacggctgccacgtggaattgctcttcctccgctacatctcggactgggacctaga
ccctggccgctgctaccgcgtcacctggttcacctcctggagcccctgctacgactgtgcccgacatgtggccgactttctgc
gagggaaccccaacctcagtctgaggatcttcaccgcgcgcctctacttctgtgaggaccgcaaggctgagcccgaggggctg
cggcggctgcaccgcgccggggtgcaaatagccatcatgaccttcaaagattatttttactgctggaatactttttgtagaaaa
ccacgaaagaactttcaaagcctgggaagggctgcatgaaaattcagttcgtctctccagacagcttcggcgcatccttttgc
ccctgtatgaggttgatgacttacgagacgcatttcgtactttgggactt

Primers for ZF-AID constructs

ZFP-F	ATCGGCTAGCCCCAGAGTGAGAACCGGT
ZFP-R-4AA	ccggttcatcaagaggctgtcAGAACCACCACCGGATCCTTTTTCACCTGTATG
ZFP-R-4AA2	ccggttcatcaagaggctgtcAGAACCACGCAGGGATCCTTTTTCACCTGTATG
ZFP-R-8AA	ctgtcAGTCGACCCCAGACCACCACCAGAGGATCCTTTTTCACCTGTATG
ZFP-R-11AA	gtcACCACCACCACCAGAACCACCACCACCAGAACCGGATCCTTTTTCACCTGTATG
ZFP-R-Hind3	atcgaagcttGGATCCTTTTTCACCTGTATG
AID-F-Nhe1	ATCGGCTAGCgacagcctcttgatgaaccg
AID-F-4AA	TACAGGTGAAAAAGGATCCGGTGGTGGTTCTgacagcctcttgatgaaccg
AID-F-4AA2	TACAGGTGAAAAAGGATCCCTGCGTGGTTCTgacagcctcttgatgaaccg
AID-F-8AA	AGGATCCTCTGGTGGTGGTCTGGGGTCGACTgacagcctcttgatgaaccg
AID-F-11AA	GATCCGGTTCTGGTGGTGGTGGTTCTGGTGGTGGTGGTgacagcctcttgatgaaccg
AID-R	atcgaagcttaaagtcccaaagtacgaaatgcg

Supplementary Note 2

TALE-AID constructs and the PCR primer sequences

TALE-AID full sequence: TALE N-terminus is in Blue, TALE central repeating domain is in Red, TALE-C terminus is in Green. Linker sequence is in Brown. AID coding sequence is un-capitalized in Black.

ATGTCGCGGACCCGGCTCCCTTCCCCACCCGCACCCAGCCCAGCGTTTTCGGCCGACTCGTTCTCAGACCTGCTTAGGCAGTT
CGACCCCTCACTGTTTAACACATCGTTGTTCGACTCCCTTCCTCCGTTTGGGGCGCACCATACGGAGGCGGCCACCGGGGAGT
GGGATGAGGTGCAGTCGGGATTGAGAGCTGCGGATGCACCACCCCCAACCATGCGGGTGGCCGTCACCGCTGCCCGACCGCCG
AGGGCGAAGCCCGCACCAAGGCGGAGGGCAGCGCAACCGTCCGACGCAAGCCCCGCAGCGCAAGTAGATTTGAGAACTTTGGG
ATATTCACAGCAGCAGCAGGAAAAGATCAAGCCCAAAGTGAGGTCGACAGTCGCGCAGCATCACGAAGCGCTGGTGGGTCATG
GGTTTACACATGCCCACATCGTAGCCTTGTCGCAGCACCCTGCAGCCCTTGGCACGGTCGCCGTCAAGTACCAGGACATGATT
GCGGCGTTGCCGGAAGCCACACATGAGGCGATCGTCGGTGTGGGGAAACAGTGGAGCGGAGCCCGAGCGCTTGAGGCCCTGTT
GACGGTCGCGGGAGAGCTGAGAGGGCCTCCCCTTCAGCTGGACACGGGCCAGTTGCTGAAGATCGCGAAGCGGGGAGGAGTCA
CGGCGGTCGAGGCGGTGCACGCGTGGCGCAATGCGCTCACGGGAGCACCCCTCAACCTGACCCCAGAGCAGGTCGTGGCAATC
GCCTCCAACATTGGCGGGAAACAGGCACTCGAGACTGTCCAGCGCCTGCTTCCCGTGCTGTGCCAAGCGCACGGACTCACCCC
AGAGCAGGTCGTGGCGATCGCAAGCCACGACGGAGGAAAGCAAGCCTTGGAAACAGTACAGAGGCTGTTGCCTGTGCTGTGCC
AAGCGCACGGCCTCACCCCAGAGCAGGTCGTGGCAATCGCGAGCAATAACGGCGGAAAACAGGCTTTGGAAACGGTGCAGAGG
CTCCTTCCAGTGCTGTGCCAAGCGCACGGATTAACCCCAGAGCAGGTCGTGGCAATCGCCTCCAACATTGGCGGGAAACAGGC
ACTCGAGACTGTCCAGCGCCTGCTTCCCGTGCTGTGCCAAGCGCACGGCTTAACCCCAGAGCAGGTCGTGGCGATCGCAAGCC
ACGACGGAGGAAAGCAAGCCTTGGAAACAGTACAGAGGCTGTTGCCTGTGCTGTGCCAAGCGCACGGACTTACCCCAGAGCAG
GTCGTGGCCATTGCCTCGAATGGAGGGGGCAAACAGGCGTTGGAAACCGTACAACGATTGCTGCCGGTGCTGTGCCAAGCGCA
CGGCCTTACCCCAGAGCAGGTCGTGGCGATCGCAAGCCACGACGGAGGAAAGCAAGCCTTGGAAACAGTACAGAGGCTGTTGC
CTGTGCTGTGCCAAGCGCACGGACTAACCCCAGAGCAGGTCGTGGCAATCGCCTCCAACATTGGCGGGAAACAGGCACTCGAG
ACTGTCCAGCGCCTGCTTCCCGTGCTGTGCCAAGCGCACGGGCTCACCCCAGAGCAGGTCGTGGCGATCGCAAGCCACGACGG
AGGAAAGCAAGCCTTGGAAACAGTACAGAGGCTGTTGCCTGTGCTGTGCCAAGCGCACGGGCTAACCCCAGAGCAGGTCGTGG
CCATTGCCTCGAATGGAGGGGGCAAACAGGCGTTGGAAACCGTACAACGATTGCTGCCGGTGCTGTGCCAAGCGCACGGCCTA
ACCCCAGAGCAGGTCGTGGCAATCGCCTCCAACATTGGCGGGAAACAGGCACTCGAGACTGTCCAGCGCCTGCTTCCCGTGCT
GTGCCAAGCGCACGGGTTAACCCCAGAGCAGGTCGTGGCCATTGCCTCGAATGGAGGGGGCAAACAGGCGTTGGAAACCGTAC
AACGATTGCTGCCGGTGCTGTGCCAAGCGCACGGACTCACGCCTGAGCAGGTAGTGGCTATTGCATCCAATATCGGGGGCAGA
CCCGCACTGGAGTCAATCGTGGCCCAGCTTTCGAGGCCGGACCCCGCGCTGGCCGCACTCACTAATGATCATCTTGTAGCGCT
GGCCTGCCTCGGCGGACGACCCGCCTTGGATGCGGTGAAGAAGGGGCTCCCGCACGCGCCTGCATTGATTAAGCGGACCAACA
GAAGGATTCCCGAGAGGACATCACATCGAGTGGCAGATCACGCGCAAGTGGTCCGCGTGCTCGGATTCTTCCAGTGTCACTCC
CACCCCGCACAAGCGTTCGATGACGCCATGACTCAATTTGGTATGTCGAGACACGGACTGCTGCAGCTCTTTCGTAGAGTCGG
TGTCACAGAACTCGAGGCCCGCTCGGGCACACTGCCTCCCGCCTCCCAGCGGTGGGACAGGATTCTCCAAGCGAGCGGTATGA

AACGCGCGAAGCCTTCACCTACGTCAACTCAGACACCTGACCAGGCGAGCCTTCATGCGTTCGCAGACTCGCTGGAGAGGGAT
TTGGACGCGCCCTCGCCCATGCATGAAGGGGACCAAACTCGCGCGTCAGCTAGCCCCAAGAAGAAGAGAAAGGTGGAGGCCAG
Cgacagcctcttgatgaaccggaggaagtttctttaccaattcaaaaatgtccgctgggctaagggtcggcgtgagacctacc
tgtgctacgtagtgaagaggcgtgacagtgctacatccttttcactggactttggttatcttcgcaataagaacggctgccac
gtggaattgctcttcctccgctacatctcggactgggacctagaccctggccgctgctaccgcgtcacctggttcacctcctg
gagcccctgctacgactgtgcccgacatgtggccgactttctgcgagggaaccccaacctcagtctgaggatcttcaccgcgc
gcctctacttctgtgaggaccgcaaggctgagcccgaggggctgcggcggctgcaccgcgccggggtgcaaatagccatcatg
accttcaaagattattttactgctggaatacttttgtagaaaaccacgaaagaactttcaaagcctgggaagggctgcatga
aaattcagttcgtctctccagacagcttcggcgcatccttttgcccctgtatgaggttgatgacttacgagacgcatttcgta
ctttgggactttga

Primer sequences

TAL-F-ssp1	TGGCAAATATTCTGAAATGAGCTGTTGACAATTAATCATCCGGTCCGTATAATCTGTGGAAT TGTGAGCGGATAACAATTTCACACAAAGAGGAGAAAGGTACCATGTCGCGGACCCGGCTCCC
TAL-C1-Nhe1	TGGGGCTAGCTGACGCGCGAGTTTGGTCCC
TAL-C2-Nhe1	TCTTGGGGCTAGCGCGGGAGGCAGTGTGCCCGA
TAL-C3-Nhe1	TCTTGGGGCTAGCTGCCACTCGATGTGATGTCCTCTCGGGAATCCT
TAL-C4-Nhe1	TCTTGGGGCTAGCGCGGCCAGCGCGGGGTCCG
TAL-C5-Nhe1	TCTTGGGGCTAGCCTCCAGTGCGGGTCTGCC
AID-R	atcgaagcttaaagtcccaaagtacgaaatgcg

Supplementary Note 3

APOBECs and PCR primer sequences

APOBEC1 sequence:

ACTTCTGAAAAAGGTCCATCTACTGGTGATCCTACTCTGCGTCGTCGTATTGAACCGTGGGAATTTGACGTGTTCTACGACCC
ACGCGAACTGCGTAAAGAGGCTTGCCTGCTGTACGAAATCAAATGGGGTATGTCTCGCAAAATTTGGCGCTCCAGCGGTAAAA
ACACCACTAACCACGTTGAAGTCAACTTCATCAAAAAGTTCACCTCTGAACGCGACTTCCACCCGTCCATGTCTTGTTCTATC
ACCTGGTTCCTGTCTTGGAGCCCGTGCTGGGAGTGCTCCCAAGCCATCCGCGAATTCCTGTCTCGTCACCCGGGTGTAACGCT
GGTGATCTATGTCGCCCGTCTGTTCTGGCATATGGATCAGCAAAACCGTCAGGGTCTGCGTGATCTGGTGAACAGCGGCGTCA
CGATCCAGATCATGCGTGCATCCGAATATTACCATTGCTGGCGTAACTTCGTAAACTACCCTCCGGGTGATGAAGCGCACTGG
CCGCAATACCCGCCGCTGTGGATGATGCTGTACGCTCTGGAGCTGCATTGCATCATCCTGTCTCTGCCACCGTGCCTGAAAAT
TTCCCGCCGTTGGCAGAACCATCTGACCTTCTTCCGTCTGCATCTGCAGAACTGTCACTACCAGACTATCCCGCCTCACATCC
TGCTGGCTACTGGCCTGATCCATCCGTCTGTTGCGTGGCGC

APOBEC3F sequence

AAACCGCATTTTCGTAACACCGTTGAGCGTATGTATCGTGACACTTTCTCTTACAACTTCTACAACCGTCCGATCCTGTCTCG
CCGCAACACCGTGTGGCTGTGTTATGAAGTTAAAACCAAAGGCCCGTCTCGTCCGCGTCTGGACGCGAAGATCTTCCGTGGCC
AGGTACCGCGTTCCTTTATTCGTGCGCCGTTTCAGGTGCTGTCTAGCCCGTTCGGCCAGTGTGCACCGCCGCACGGTACGGCG
CAGGTTCAATGGCCTCCGCAGCTGACTGCCGGTCGCGAGCAGGGTCGTCCG

APOBEC3G 2K3A sequence

GAAATTCTGCGTCACTCTATGGACCCGCCAACTTTTACTTTCAACTTCAACAATGAACCGTGGGTCCGTGGCCGTCACGAGAC
TTACCTGTGCTACGAGGTGGAGCGTATGCACAATGATACCTGGGTGAAACTGAACCAGCGTCGCGGTTTCCTGGCTAACCAGG
CTCCGCACAAACACGGCTTCCTGGAGGGCCGTCACGCTGAACTGTGCTTCCTGGATGTTATTCCTTTCTGGAAACTGGACCTG
GACCAAGATTATCGTGTAACTTGCTTCACTAGCTGGAGCCCATGCTTCAGCTGCGCACAGGAAATGGCCAAGTTCATTTCTAA
AAACAAACATGTTTCTCTGTGTATCAAGACTGCTCGCATCTATGATGACCAGGGCCGTGCTCAGGAAGGCCTGCGTACTCTGG
CGGAAGCAGGTGCTAAAATTAGCATCATGACTTACAGCGAATTCAAACACTGCTGGGACACCTTCGTGGACCACCAGGGTGCG
CCTTTCCAGCCTTGGGATGGTCTGGATGAACACTCTCAGGACCTGTCTGGTCGTCTGCGTGCGATCCTGCAGAACCAGGAAAA
T

Primers for ZF-APOBECs constructs

Homology to the vectors is in Black; linker sequence is highlighted in Green and the homology to the APOBECs sequences is in Red.

APOBEC1-F-4AA1	GAAAAGACATCTGAAGACACATACAGGTGAAAAAGGATCCGGTGGTGGTTCTACTTCTGAAAAAGGTCCATCTAC
APOBEC1-reverse	CCATGGGATCCCCCGGGCTGCAGGAATTCGATATCAAGCTTTCAGCGCCACGCAACAGAC
APOBEC3F-F-4AA1	GAAAAGACATCTGAAGACACATACAGGTGAAAAAGGATCCGGTGGTGGTTCT AAACCGCATTTTCGTAACACCGTTGAGCG
APOBEC3F-reverse	CCATGGGATCCCCCGGGCTGCAGGAATTCGATATCAAGCTTCACGGACGACCCTGCTCGC
APOBEC3G-F-4AA1	GAAAAGACATCTGAAGACACATACAGGTGAAAAAGGATCCGGTGGTGGTTCT GAAATTCTGCGTCACTCTATGGAC
APOBEC3G-reverse	CCATGGGATCCCCCGGGCTGCAGGAATTCGATATCAAGCTTTCAATTTTCCTGGTTCTGC

<u>Primers for TALE-APOBECs construct</u>

NheI cutting site is in Red and HindIII cutting site is in the Blue.

APOBEC1-F	ATCGGCTAGCCCCAAGAAGAAGAGAAAGGTGGAGGCCAGCACTTCTGAAAAAGGTCCATCTACTGGTG
APOBEC1-R	ATCGAAGCTTTCAGCGCCACGCAACAGACGGATGG
APOBEC3F-F	ATCGGCTAGCCCCAAGAAGAAGAGAAAGGTGGAGGCCAGCAAACCGCATTTTCGTAACACCGTTGAG
APOBEC3F-R	ATCGAAGCTTTCACGGACGACCCTGCTCGCGACCG
APOBEC-3G-F	ATCGGCTAGCCCCAAGAAGAAGAGAAAGGTGGAGGCCAGCGAAATTCTGCGTCACTCTATG
APOBEC-3G-R	ATCGAAGCTTTCAATTTTCCTGGTTCTGCAGGATCG

Supplementary Note 4

pTrc-Kan backbone sequence

CTCGAGGTGGTGAATGTGAAACCAGTAACGTTATACGATGTCGCAGAGTATGCCGGTGTCTCTTATCAGACCGTTTCCCGCGT
GGTGAACCAGGCCAGCCACGTTTCTGCGAAAACGCGGGAAAAAGTGGAAGCGGCGATGGCGGAGCTGAATTACATTCCCAACC
GCGTGGCACAACAACTGGCGGGCAAACAGTCGTTGCTGATTGGCGTTGCCACCTCCAGTCTGGCCCTGCACGCGCCGTCGCAA
ATTGTCGCGGCGATTAAATCTCGCGCCGATCAACTGGGTGCCAGCGTGGTGGTGTCGATGGTAGAACGAAGCGGCGTCGAAGC
CTGTAAAGCGGCGGTGCACAATCTTCTCGCGCAACGCGTCAGTGGGCTGATCATTAACTATCCGCTGGATGACCAGGATGCCA
TTGCTGTGGAAGCTGCCTGCACTAATGTTCCGGCGTTATTTCTTGATGTCTCTGACCAGACACCCATCAACAGTATTATTTTC
TCCCATGAAGACGGTACGCGACTGGGCGTGGAGCATCTGGTCGCATTGGGTCACCAGCAAATCGCGCTGTTAGCGGGCCCATT
AAGTTCTGTCTCGGCGCGTCTGCGTCTGGCTGGCTGGCATAAATATCTCACTCGCAATCAAATTCAGCCGATAGCGGAACGGG
AAGGCGACTGGAGTGCCATGTCCGGTTTTCAACAAACCATGCAAATGCTGAATGAGGGCATCGTTCCCACTGCGATGCTGGTT
GCCAACGATCAGATGGCGCTGGGCGCAATGCGCGCCATTACCGAGTCCGGGCTGCGCGTTGGTGCGGATATCTCGGTAGTGGG
ATACGACGATACCGAAGACAGCTCATGTTATATCCCGCCGTTAACCACCATCAAACAGGATTTTCGCCTGCTGGGGCAAACCA
GCGTGGACCGCTTGCTGCAACTCTCTCAGGGCCAGGCGGTGAAGGGCAATCAGCTGTTGCCCGTCTCACTGGTGAAAAGAAAA
ACCACCCTGGCACCCAATACGCAAACCGCCTCTCCCCGCGCGTTGGCCGATTCATTAATGCAGCTGGCACGACAGGTTTCCCG
ACTGGAAAGCGGGCAGTGAGCGCAACGCAATTAATGTGAGTTAGCGCGAATTGATCTGGTTTGACAGCTTATCATCGACTGCA
CGGTGCACCAATGCTTCTGGCGTCAGGCAGCCATCGGAAGCTGTGGTATGGCTGTGCAGGTCGTAAATCACTGCATAATTCGT
GTCGCTCAAGGCGCACTCCCGTTCTGGATAATGTTTTTTGCGCCGACATCATAACGGTTCTGGCAAATATTCTGAAATGAGCT
GTTGACAATTAATCATCCGGTCCGTATAATCTGTGGAATTGTGAGCGGATAACAATTTCACACAGGAAACAGACCATGTCGTA
CTACCATCACCATCACCATCACGATTACGATATCCCAACGACCGAAAACCTGTATTTTCAGGGCGCCGCTAGCCCCAGCGACT
AAGCTTGATATCGAATTCCTGCAGCCCGGGGGATCCCATGGTACGCGTGCTAGAGGCATCAAATAAAACGAAAGGCTCAGTCG
AAAGACTGGGCCTTTCGTTTTATCTGTTGTTTGTCGGTGAACGCTCTCCTGAGTAGGACAAATCCGCCGCCCTAGACCTAGGG
CGTTCGGCTGCGGCGAGCGGTATCAGCTCACTCAAAGGCGGTAATACGGTTATCCACAGAATCAGGGGATAACGCAGGAAAGA
ACATGTGAGCAAAAGGCCAGCAAAAGGCCAGGAACCGTAAAAAGGCCGCGTTGCTGGCGTTTTTCCATAGGCTCCGCCCCCCT
GACGAGCATCACAAAAATCGACGCTCAAGTCAGAGGTGGCGAAACCCGACAGGACTATAAAGATACCAGGCGTTTCCCCCTGG
AAGCTCCCTCGTGCGCTCTCCTGTTCCGACCCTGCCGCTTACCGGATACCTGTCCGCCTTTCTCCCTTCGGGAAGCGTGGCGC
TTTCTCATAGCTCACGCTGTAGGTATCTCAGTTCGGTGTAGGTCGTTCGCTCCAAGCTGGGCTGTGTGCACGAACCCCCCGTT
CAGCCCGACCGCTGCGCCTTATCCGGTAACTATCGTCTTGAGTCCAACCCGGTAAGACACGACTTATCGCCACTGGCAGCAGC
CACTGGTAACAGGATTAGCAGAGCGAGGTATGTAGGCGGTGCTACAGAGTTCTTGAAGTGGTGGCCTAACTACGGCTACACTA
GAAGGACAGTATTTGGTATCTGCGCTCTGCTGAAGCCAGTTACCTTCGGAAAAAGAGTTGGTAGCTCTTGATCCGGCAAACAA
ACCACCGCTGGTAGCGGTGGTTTTTTTGTTTGCAAGCAGCAGATTACGCGCAGAAAAAAAGGATCTCAAGAAGATCCTTTGAT
CTTTTCTACGGGGTCTGACGCTCAGTGGAACGAAAACTCACGTTAAGGGATTTTGGTCATGACTAGTGCTTGGATTCTCACCA
ATAAAAAACGCCCGGCGGCAACCGAGCGTTCTGAACAAATCCAGATGGAGTTCTGAGGTCATTACTGGATCTATCAACAGGAG

TCCAAGCGAGCTCTCGAACCCCAGAGTCCCGCTCAGAAGAACTCGTCAAGAAGGCGATAGAAGGCGATGCGCTGCGAATCGGG
AGCGGCGATACCGTAAAGCACGAGGAAGCGGTCAGCCCATTCGCCGCCAAGCTCTTCAGCAATATCACGGGTAGCCAACGCTA
TGTCCTGATAGCGGTCCGCCACACCCAGCCGGCCACAGTCGATGAATCCAGAAAAGCGGCCATTTTCCACCATGATATTCGGC
AAGCAGGCATCGCCATGGGTCACGACGAGATCCTCGCCGTCGGGCATGCGCGCCTTGAGCCTGGCGAACAGTTCGGCTGGCGC
GAGCCCCTGATGCTCTTCGTCCAGATCATCCTGATCGACAAGACCGGCTTCCATCCGAGTACGTGCTCGCTCGATGCGATGTT
TCGCTTGGTGGTCGAATGGGCAGGTAGCCGGATCAAGCGTATGCAGCCGCCGCATTGCATCAGCCATGATGGATACTTTCTCG
GCAGGAGCAAGGTGAGATGACAGGAGATCCTGCCCCGGCACTTCGCCCAATAGCAGCCAGTCCCTTCCCGCTTCAGTGACAAC
GTCGAGCACAGCTGCGCAAGGAACGCCCGTCGTGGCCAGCCACGATAGCCGCGCTGCCTCGTCCTGCAGTTCATTCAGGGCAC
CGGACAGGTCGGTCTTGACAAAAAGAACCGGGCGCCCCTGCGCTGACAGCCGGAACACGGCGGCATCAGAGCAGCCGATTGTC
TGTTGTGCCCAGTCATAGCCGAATAGCCTCTCCACCCAAGCGGCCGGAGAACCTGCGTGCAATCCATCTTGTTCAATCATGCG
AAACGATCCTCATCCTGTCTCTTGATCAGATCTTGAT

Supplementary Note 5

pL-tetO promoter and PCR primers

CTCGAGTCCCTATCAGTGATAGAGATTGACATCCCTATCAGTGATAGAGATACTGAGCACATCAGCAGGACGCACTGACCGAA
TTCATTAAAGAGGAGAAAGGTACC

pL-tetO-5	ATCGCTCGAGTCCCTATCAGTGATAGAGATTGACATCC
pL-tetO-3	ACTCTGGGGCTAgcCATGGTACCTTTCTCCTCTTTAATG

Supplementary Note 6

GFP reporter cassette, PCR primer sequences and recombineering oligos

GFP reporter cassette

Start codon is in Blue and the ZFP binding site is highlighted in Yellow, GFP coding sequence is in Green.

CGCGAAATTAATACGACTCACTATAGGGAGACCACAACGGTTTCCCTCTAGAAATAATTTTGTTTAACTTTAAGAAGGAGATA
TACATATGCGGGGTTCTGCCGCAGTGGGTATGGCTAGCATGACTGGTGGACAGCAAATGGGTCGGGATCTGTACGACGATGAC
GATAAGGATCGATGGGGATCCGAATTCGCCACCATGGTGAGCAAGGGCGAGGAGCTGTTCACCGGGGTGGTGCCCATCCTGGT
CGAGCTGGACGGCGACGTAAACGGCCACAAGTTCAGCGTGTCCGGCGAGGGCGAGGGCGATGCCACCTACGGCAAGCTGACCC
TGAAGTTCATCTGCACCACCGGCAAGCTGCCCGTGCCCTGGCCCACCCTCGTGACCACCTTGACCTACGGCGTGCAGTGCTTC
GCCCGCTACCCCGACCACATGAAGCAGCACGACTTCTTCAAGTCCGCCATGCCCGAAGGCTACGTCCAGGAGCGCACCATCTT
CTTCAAGGACGACGGCAACTACAAGACCCGCGCCGAGGTGAAGTTCGAGGGCGACACCCTGGTGAACCGCATCGAGCTGAAGG
GCATCGACTTCAAGGAGGACGGCAACATCCTGGGGCACAAGCTGGAGTACAACTACAACAGCCACAAGGTCTATATCACCGCC
GACAAGCAGAAGAACGGCATCAAGGTGAACTTCAAGACCCGCCACAACATCGAGGACGGCAGCGTGCAGCTCGCCGACCACTA
CCAGCAGAACACCCCCATCGGCGACGGCCCCGTGCTGCTGCCCGACAACCACTACCTGAGCACCCAGTCCGCCCTGAGCAAAG
ACCCCAACGAGAAGCGCGATCACATGGTCCTGCTGGAGTTCGTGACCGCCGCCGGGATCACTCTCGGCATGGACGAGCTGTAC
AAGTAACTCGAGAAGCTTGATCCGGCTGCTAACAAAGCCCGAAAGGAAGCTGAGTTGGCTGCTGCCACCGCTGAGCAATAACT
AGCATAACCCCTTGGGGCCTCTAAACGGGTCTTGAGGGGTTTTTTGCTGAAAGGAGGAACTATATCCGGATCTGGCGT

Primers for reporter integration

5'-galk-gfp	atcaaaccgtgatcagttgtgcaccacgcgatgaccgtaaCGCGAAATTAATACGACTCAC
3'-gfp-galk	gtcgagctgattttcataatcggctgccatcacgcgaactACGCCAGATCCGGATATAGTTC

Oligo designed for reporter modification

The start codon position (ACG/ATG) is in Red, and the ZF/TALE binding site is highlighted in yellow and blue respectively. * is the phosphothioester bond.

ZFP-ACG	C*T*CTAGAAATAATTTTGTTTAACTTTAAGAAGGAGATATACAAACGCGGGGTTCTGCC GCAGTGGGTATGGCTAGCATGACTGGTGGA*C*A
TAL-ACG-3bp-spacer	TAATTTTGTTTAACTTTAAGAAGGAGATATACATACGAcGGGAAGAATCGTGAGTATGGC

	TAGCATGACTGGTGGACAGCAAATGGGTCG
TAL-ACG-6bp-spacer	TTTGTTTAACTTTAAGAAGGAGATATACATACGATTAGGGGAAGAATCGTGAGTATGGCTAGCATGACTGGTGGACAGCAAATGGGT
TAL-ACG-9bp-spacer	TTTGTTTAACTTTAAGAAGGAGATATACATACGATTAGTCTGGGAAGAATCGTGAGTATGGCTAGCATGACTGGTGGACAGCAAATGGGT
TAL-ACG-12bp-spacer	ACTTTAAGAAGGAGATATACATACGATTAGTCTGTTGGGAAGAATCGTGAGTATGGCTAGCATGACTGGTGGACAGCAAATGGGTCGGGA
TAL-ACG-15bp-spacer	TTGTTTAACTTTAAGAAGGAGATATACATACGATTAGTCTGTTTACGGGAAGAATCGTGAGTATGGCTAGCATGACTGGTGGACAGCAAA
APOBEC1-ACG-ZFP	C*T*C*TAGAAATAATTTTGTTTAACTTTAAGAAGGAGATATACAAACGAAACAACAAGCCGCAGTGGGTATGGCTAGCATGACTGGTG*G*A*C
APOBEC3F-ACG-ZFP	C*T*C*TAGAAATAATTTTGTTTAACTTTAAGAAGGAGATATACATACGAAACAACAAGCCGCAGTGGGTATGGCTAGCATGACTGGTG*G*A*C
APOBEC3G-ACG-ZFP	C*T*C*TAGAAATAATTTTGTTTAACTTTAAGAAGGAGATATACACACGCAACAACAAGCCGCAGTGGGTATGGCTAGCATGACTGGTG*G*A*C
APOBEC1-ACG-TAL	C*C*TCTAGAAATAATTTTGTTTAACTTTAAGAAGGAGATATACAAACGATTAGTCTGGGAAGAATCGTGAGTATGGCTAGCATGACTGG*T*G
APOBEC3F-ACG-TAL	C*C*TCTAGAAATAATTTTGTTTAACTTTAAGAAGGAGATATACATACGATTAGTCTGGGAAGAATCGTGAGTATGGCTAGCATGACTGG*T*G
APOBEC3G-ACG-TAL	C*T*CTAGAAATAATTTTGTTTAACTTTAAGAAGGAGATATACACACGCTTAGTCTGGGAAGAATCGTGAGTATGGCTAGCATGACTGG*T*G
ATG-NNCCAA-ZFP	A*A*TTTTGTTTAACTTTAAGAAGGAGATATACAAATGANNCAATTATTACTGCCGCAGTGTGGTATGGCTAGCATGACTGGTGGACAGC*A*A

Supplementary Note 7

Zeocin resistance cassette and PCR primer sequences

TTTGCTGGCCTTTTGCTCACATGTGTGCTGGGCCCAGCCGGCCAGATCTGAGCTCGCGGCCGCGATATCGCTAGCTCGAGCAC
GTGTTGACAATTAATCATCGGCATAGTATATCGGCATAGTATAATACGACAAGGTGAGGAACTAAACCATGGCCAAGTTGACC
AGTGCCGTTCCGGTGCTCACCGCGCGCGACGTCGCCGGAGCGGTCGAGTTCTGGACCGACCGGCTCGGGTTCTCCCGGGACTT
CGTGGAGGACGACTTCGCCGGTGTGGTCCGGGACGACGTGACCCTGTTCATCAGCGCGGTCCAGGACCAGGTGGTGCCGGACA
ACACCCTGGCCTGGGTGTGGGTGCGCGGCCTGGACGAGCTGTACGCCGAGTGGTCGGAGGTCGTGTCCACGAACTTCCGGGAC
GCCTCCGGGCCGGCCATGACCGAGATCGGCGAGCAGCCGTGGGGGCGGGAGTTCGCCCTGCGCGACCCGGCCGGCAACTGCGT
GCACTTCGTGGCCGAGGAGCAGGACTGAGAATTCCCGGGGATCCTCTAGAGTCGACCTGCAGGCATGCAAGCTTGGCACTGGC
CGTCGTTTTACAACGTCGTGACTGGGAAAACCCTGGCGTTACCCAACTTAATCGCCTTGCAGCACATCCCCCTTTCGCCAGCT
GGCGTAATAGCGAAGAGGCCCGCACCGATCGCCCTTCCCAACAGTTGCGCAGCCTGAATGGCGAATGGCGCCTGATGCGGTAT
TTTCTCCTTACGCATCTGTGCGGTATTTCACACCGCATATATGGTGCACTCTCAGTACAATCTGCTCTGATGCCGCATAGTTA
AGCCAGCCCCGACACCCGCCAACACCCGC

5'ung-zeo	ATGGCTAACGAATTAACCTGGCATGACGTGCTGGCTGAAGCTTTTGCTGGCCTTTTGCTC
3'zeo-ung	TTACTCACTCTCTGCCGGTAATACTGGCATCCAGTCAATCGTCAGCGGGTGTTGGCGGGT

Supplementary Note 8

Test of targeted deamination frequency on a reporter with two ZF binding sites

Although AID was observed to function as a monomer, it has also been postulated that AID forms homodimers and homotetramers based on structural modeling with homologous cytidine deaminases. Having shown that a single ZF binding site was sufficient for ZF-AID editing **(Fig. 1b)**, we next sought to test whether we can increase the targeted deamination frequency by adding another zinc finger binding site. This would facilitate dimerization of AID, if it functions as a dimer. To this end, we first sought to modify the reporter by adding two ZF

binding sites flanking the targeting site (the broken start codon, ACG) while ensuring that the modifications would not compromise the expression of the GFP protein. Four different modified GFP reporters were investigated **(Supplementary Fig. 2a),** however only one reporter in which an additional ZF binding sequence (5'GCCGACGTG3' in the bottom strand) lay 14bp upstream of the start codon did not compromise the translation efficiency **(Supplementary Fig. 2b).** Therefore, we further modified this reporter by mutating its start codon to ACG with MAGE and used it to conduct further studies. Interestingly, induction of ZF-AID led to similar GFP rescue frequency (0.1%) on the dimer reporter as the one with a single ZF binding site (0.12%) **(Supplementary Fig. 2c),** indicating that the targeting a single copy of ZF-AID at the targeting site is sufficient to exert deaminase activity in the cell. Future experiment with symmetrical zinc finger DNA binding sites is needed to substantiate this conclusion. Also, test the deamination frequency of ZF-AID with a mutated dimerization interface might help determine the *stoichiometry* of the functional ZF-AIDs.

Supplementary Note 9

Time course of the targeted deamination frequency and investigation into secondary mutations:

The GFP rescue efficiency increased over the time and reached the peak after ZF-AID induction for 30 hours. Interestingly, prolonged induction of ZF-AIDs led to decreases in GFP expression **(Supplementary Fig. 3a)** after 30 hours. This decrease was also observed with the prolonged induction of TALE-C3-AID **(Supplementary Fig. 3b).** To determine the reason for this decrease, we first isolated single colonies with rescued GFP expression, and then checked for GFP signal again following a second round of ZF-8aa-AID induction **(Supplementary Fig. 3c)**. Expression of ZF-8aa-AID led to 1% of GFP+ cells losing GFP signal after 10 hours, whereas no detectable GFP loss was observed in the uninduced population. An ATG/TAC →ATA/TAT transition at the start codon accounted for 95% (19/20) of this loss **(Supplementary Fig. 3d),** suggesting that the decline of GFP signal was due to the accumulation of secondary mutations close to the targeted site. Future investigation is needed to achieve sequence specific targeting with single nucleotide resolution. Also, this observation suggests that ZFP-AIDs can edit cytosines on both sense and antisense strands effectively.

Supplementary Note 10

Investigation on the effect of target gene transcription

Recombination-based genome editing is reportedly more efficient if the target locus is transcriptionally active and transcription is required for AID's activity in vitro and in vivo, presumably by exposing the non-transcribed strand as ssDNA substrate for AID. We sought to determine whether transcription of the targeted site is necessary for ZF-AID function. In our reporter systems described in the manuscript, GFP and targeted deaminase were both induced by IPTG, with the result that GFP is inevitably transcriptionally active during deaminase targeting. To investigate the editing frequency on a non-active target locus, we built an orthogonal inducible expression system. GFP is transcribed from the T7 promoter **(Supplementary Fig. 4a)** using T7 polymerases transcribed from pTac promoter and induced by Isopropyl-β-D-thio-galactoside (IPTG) (100uM). ZF-AID expression was controlled by p_L-tetO **(Supplementary Fig. 4b),** a modified version of the native Phage P_L promoter containing two TetR operator sites, so that the transcription is inhibited by the TetR protein (constitutively expressed in the EcNR2 strain) and induced by anhydrotetracycline (aTc) (30ng/ml). We then induced ZF-AID expression for 10 hours either with or without simultaneous induction of GFP. Under both conditions the cell culture was diluted 1:1000 in fresh LB media and cultured in the

absence of aTc., after which GFP was re-induced in all cells with IPTG (100uM) for 7 hours to estimate target site editing frequency **(Supplementary Fig. 4c)**.

Induction of ZF-AID and GFP simultaneously led to robust GFP rescue (5.16**%**) **(Supplementary Fig. 4d).** In contrast, significantly lower GFP rescue frequency (0.74%) was observed when ZF-AID alone was induced **(Supplementary Fig. 4d)** alone (t-test, two-tailed, p<0.0001, n=4). The seven-fold enhancement of GFP rescue in the presence of transcription is probably a lower limit, since residual ZF-AID from the aTc induction would still be able to act on the transcribed GFP locus during the test for GFP expression. This result suggests that targeted deaminase genome editing occurs with increased efficiency if the target locus is in an active.

Supplementary Note 11

GFP and GAPDH amplification and sequence primer sequences

Amplification-GFP-5	Cgtttgcgcgcagtcagcgatatccattttcgcgaatccg
Ampliciation-GFP-3	CGCAGTTACAGCCTACAAACTGGTTTTCTGCTTC
Sequencing-GFP-f	Atgagtctgaaagaaaaaacacaatc
Sequencing-GFP-r	TGACCGTTAAGCGCGATTTG
Amplification-GAPDH-5	Tatttacagtcttaatgagtgaaagaggcggagg
Amplification-GAPDH-3	Gccatcctggtctaagcttggaaagg
Sequencing-GAPDH-f	Aggcggaggtttttcctccgcctgtgcgcg
Sequencing-GAPDH-r	Atcaattttcatccgaacgttcc

Supplementary Note 12

Next generation adaptor and PCR primer sequences

Adaptor1	PE-A1-F	TACACTCTTTCCCTACACGACGCTCTTCCGATCTac*T
	PE-A1-R	/5Phos/gtAGATCGGAAGAGCGGTTCAGCAGGAATGCCGAG
Adaptor2	PE-A2-F	TACACTCTTTCCCTACACGACGCTCTTCCGATCTtg*T
	PE-A2-R	/5Phos/caAGATCGGAAGAGCGGTTCAGCAGGAATGCCGAG
Adaptor3	PE-A3-F	TACACTCTTTCCCTACACGACGCTCTTCCGATCT tc*T
	PE-A3-R	/5Phos/gaAGATCGGAAGAGCGGTTCAGCAGGAATGCCGAG
Adaptor4	PE-A4-F	TACACTCTTTCCCTACACGACGCTCTTCCGATCT ga*T
	PE-A4-R	/5Phos/tcAGATCGGAAGAGCGGTTCAGCAGGAATGCCGAG
Adaptor5	PE-A5-F	TACACTCTTTCCCTACACGACGCTCTTCCGATCTag*T
	PE-A5-R	/5Phos/ctAGATCGGAAGAGCGGTTCAGCAGGAATGCCGAG
Adaptor6	PE-A6-F	TACACTCTTTCCCTACACGACGCTCTTCCGATCTgt*T
	PE-A6-R	/5Phos/acAGATCGGAAGAGCGGTTCAGCAGGAATGCCGAG
Adaptor7	PE-A7-F	TACACTCTTTCCCTACACGACGCTCTTCCGATCT ct*T

	PE-A7-R	/5Phos/agAGATCGGAAGAGCGGTTCAGCAGGAATGCCGAG
Adaptor8	PE-A8-F	TACACTCTTTCCCTACACGACGCTCTTCCGATCT ca*T
	PE-A8-R	/5Phos/tgAGATCGGAAGAGCGGTTCAGCAGGAATGCCGAG
PCR primers	PE-PCR-1	AATGATACGGCGACCACCGAGATCTACACTCTTTCCCTACACGAC
	PE-PCR-2	CAAGCAGAAGACGGCATACGAGATCGGTCTCGGCATTCCTGCTGAACC

Supplementary Note 13

Human GFP-ACG reporter sequence and genotyping primers

The pEF-1α promoter sequence is in Blue, the GFP ORF is in Green and the IRES is highlighted in Gray and the mcherry ORF is in Red. Of note, the barcode sequence is highlighted in Yellow.

TGCAAAGATGGATAAAGTTTTAAACAGAGAGGAATCTTTGCAGCTAATGGACCTTCTAGGTCTTGAAAGGAGTGGGAATTGGC
TCCGGTGCCCGTCAGTGGGCAGAGCGCACATCGCCCACAGTCCCCGAGAAGTTGGGGGGAGGGGTCGGCAATTGAACCGGTGC
CTAGAGAAGGTGGCGCGGGGTAAACTGGGAAAGTGATGTCGTGTACTGGCTCCGCCTTTTTCCCGAGGGTGGGGGAGAACCGT
ATATAAGTGCAGTAGTCGCCGTGAACGTTCTTTTTCGCAACGGGTTTGCCGCCAGAACACAGGTAAGTGCCGTGTGTGGTTCC
CGCGGGCCTGGCCTCTTTACGGGTTATGGCCCTTGCGTGCCTTGAATTACTTCCACTGGCTGCAGTACGTGATTCTTGATCCC
GAGCTTCGGGTTGGAAGTGGGTGGGAGAGTTCGAGGCCTTGCGCTTAAGGAGCCCCTTCGCCTCGTGCTTGAGTTGAGGCCTG
GCCTGGGCGCTGGGGCCGCCGCGTGCGAATCTGGTGGCACCTTCGCGCCTGTCTCGCTGCTTTCGATAAGTCTCTAGCCATTT
AAAATTTTTGATGACCTGCTGCGACGCTTTTTTTCTGGCAAGATAGTCTTGTAAATGCGGGCCAAGATCTGCACACTGGTATT
TCGGTTTTTGGGGCCGCGGGCGGCGACGGGGCCCGTGCGTCCCAGCGCACATGTTCGGCGAGGCGGGGCCTGCGAGCGCGGCC
ACCGAGAATCGGACGGGGGTAGTCTCAAGCTGGCCGGCCTGCTCTGGTGCCTGGCCTCGCGCCGCCGTGTATCGCCCCGCCCT
GGGCGGCAAGGCTGGCCCGGTCGGCACCAGTTGCGTGAGCGGAAAGATGGCCGCTTCCCGGCCCTGCTGCAGGGAGCTCAAAA
TGGAGGACGCGGCGCTCGGGAGAGCGGGCGGGTGAGTCACCCACACAAAGGAAAAGGGCCTTTCCGTCCTCAGCCGTCGCTTC
ATGTGACTCCACGGAGTACCGGGCGCCGTCCAGGCACCTCGATTAGTTCTCGAGCTTTTGGAGTACGTCGTCTTTAGGTTGGG
GGGAGGGGTTTTATGCGATGGAGTTTCCCCACACTGAGTGGGTGGAGACTGAAGTTAGGCCAGCTTGGCACTTGATGTAATTC
TCCTTGGAATTTGCCCTTTTTGAGTTTGGATCTTGGTTCATTCTCAAGCCTCAGACAGTGGTTCAAAGTTTTTTTCTTCCATT
TCAGGTGTCGTGACGTACGHHHHHHHHTCCAGTAGCAGACCTACGGCCACCACGCGGGGTTCTGCCGCAGTGGATCGATGGGG
ATCCGAATTCGCCACCGTGAGCAAGGGCGAGGAGCTGTTCACCGGGGTGGTGCCCATCCTGGTCGAGCTGGACGGCGACGTAA
ACGGCCACAAGTTCAGCGTGTCCGGCGAGGGCGAGGGCGATGCCACCTACGGCAAGCTGACCCTGAAGTTCATCTGCACCACC
GGCAAGCTGCCCGTGCCCTGGCCCACCCTCGTGACCACCCTGACCTACGGCGTGCAGTGCTTCAGCCGCTACCCCGACCACAT
GAAGCAGCACGACTTCTTCAAGTCCGCCATGCCCGAAGGCTACGTCCAGGAGCGCACCATCTTCTTCAAGGACGACGGCAACT
ACAAGACCCGCGCCGAGGTGAAGTTCGAGGGCGACACCCTGGTGAACCGCATCGAGCTGAAGGGCATCGACTTCAAGGAGGAC
GGCAACATCCTGGGGCACAAGCTGGAGTACAACTACAACAGCCACAACGTCTATATCATGGCCGACAAGCAGAAGAACGGCAT
CAAGGTGAACTTCAAGATCCGCCACAACATCGAGGACGGCAGCGTGCAGCTCGCCGACCACTACCAGCAGAACACCCCCATCG
GCGACGGCCCCGTGCTGCTGCCCGACAACCACTACCTGAGCACCCAGTCCGCCCTGAGCAAAGACCCCAACGAGAAGCGCGAT
CACATGGTCCTGCTGGAGTTCGTGACCGCCGCCGGGATCACTCTCGGCATGGACGAGCTGTACAAGTAAGGCGCGCCCCCCCC
TAACGTTACTGGCCGAAGCCGCTTGGAATAAGGCCGGTGTGCGTTTGTCTATATGTTATTTTCCACCATATTGCCGTCTTTTG
GCAATGTGAGGGCCCGGAAACCTGGCCCTGTCTTCTTGACGAGCATTCCTAGGGGTCTTTCCCCTCTCGCCAAAGGAATGCAA
GGTCTGTTGAATGTCGTGAAGGAAGCAGTTCCTCTGGAAGCTTCTTGAAGACAAACAACGTCTGTAGCGACCCTTTGCAGGCA
GCGGAACCCCCCACCTGGCGACAGGTGCCTCTGCGGCCAAAAGCCACGTGTATAAGATACACCTGCAAAGGCGGCACAACCCC
AGTGCCACGTTGTGAGTTGGATAGTTGTGGAAAGAGTCAAATGGCTCTCCTCAAGCGTATTCAACAAGGGGCTGAAGGATGCC
CAGAAGGTACCCCATTGTATGGGATCTGATCTGGGGCCTCGGTGCACATGCTTTACATGTGTTTAGTCGAGGTTAAAAAACGT
CTAGGCCCCCCGAACCACGGGGACGTGGTTTTCCTTTGAAAAACACGATGATAATATGGCCACAACCATGGTGAGCAAGGGCG
AGGAGGATAACATGGCCATCATCAAGGAGTTCATGCGCTTCAAGGTGCACATGGAGGGCTCCGTGAACGGCCACGAGTTCGAG
ATCGAGGGCGAGGGCGAGGGCCGCCCCTACGAGGGCACCCAGACCGCCAAGCTGAAGGTGACCAAGGGTGGCCCCCTGCCCTT
CGCCTGGGACATCCTGTCCCCTCAGTTCATGTACGGCTCCAAGGCCTACGTGAAGCACCCCGCCGACATCCCCGACTACTTGA
AGCTGTCCTTCCCCGAGGGCTTCAAGTGGGAGCGCGTGATGAACTTCGAGGACGGCGGCGTGGTGACCGTGACCCAGGACTCC
TCCCTGCAGGACGGCGAGTTCATCTACAAGGTGAAGCTGCGCGGCACCAACTTCCCCTCCGACGGCCCCGTAATGCAGAAGAA
GACCATGGGCTGGGAGGCCTCCTCCGAGCGGATGTACCCCGAGGACGGCGCCCTGAAGGGCGAGATCAAGCAGAGGCTGAAGC
TGAAGGACGGCGGCCACTACGACGCTGAGGTCAAGACCACCTACAAGGCCAAGAAGCCCGTGCAGCTGCCCGGCGCCTACAAC

GTCAACATCAAGTTGGACATCACCTCCCACAACGAGGACTACACCATCGTGGAACAGTACGAACGCGCCGAGGGCCGCCACTC
CACCGGCGGCATGGACGAGCTGTACAAGTAA

Supplementary Note 14

UGI encoding sequence and primers

UGI encoding sequence: NLS is highlighted in Yellow.

ATGACAAATCTGAGCGATATTATAGAAAAAGAGACTGGTAAACAGCTCGTGATTCAAGAGAGTATCCTTATGCTGCCTGAGGA
AGTGGAAGAAGTTATCGGCAATAAACCCGAGTCCGACATTCTGGTGCACACGGCGTATGATGAAAGCACCGACGAAAATGTGA
TGCTGCTTACTAGCGACGCTCCAGAGTACAAGCCATGGGCCCTGGTGATTCAAGACAGTAACGGAGAGAATAAGATCAAAATG
CTCTCCGGACTCAGATCTCGAGCTGATCCAAAAAAGAAGAGAAAGGTAGATCCAAAAAAGAAGAGAAAGGTAGATCCAAAAAA
GAAGAGAAAGGTA

BsiWI-UGI	TAGGGGCGTACGGCCACCATGACAAATCTGAGCGATATTATA
XhoI-UGI	TCAGCTCGAGATCTGAGTCCGGAGAGCATTTTGATCTTATTCTC

Supplementary Note 15

ZF-AID-NLS/ZFP-AID$^{\Delta NES}$ sequences and primers

ZF-AID sequence: The NLS is highlighted in Yellow. ZF is in Red, the linker is in Green and the deaminase is in Blue. Of note, the nucleus export signal (NES) highlighted in Gray at the C-terminus of the deaminase was missing in ZF- **AID$^{\Delta NES}$**.

ATGGCTAGCCCCAGAGTGAGAACCGGTTCTAAGACACCTCCCCACGAGAGGCCTTTTCAGTGTAGAATTTGTATGCGTAATTT
TTCTAGGTCCGATGTGCTGGCCAATCACACAAGGACTCACACTGGTGAAAAGCCCTTCCAATGTAGAATTTGTATGCGCAATT
TTTCTCAATCTTCTACTCTGACTAGACATCTGAGGACCCACACAGGCGAAAAGCCTTTCCAGTGCAGAATTTGTATGAGAAAT
TTTTCTGAAAGACAGGGTCTGAAAAGACATCTGAAGACACATACAGGTGAAAAAGGATCCTCTGGTGGTGGTCTGGGTTCTAC
TGACAGCCTCTTGATGAACCGGAGGAAGTTTCTTTACCAATTCAAAAATGTCCGCTGGGCTAAGGGTCGGCGTGAGACCTACC
TGTGCTACGTAGTGAAGAGGCGTGACAGTGCTACATCCTTTTCACTGGACTTTGGTTATCTTCGCAATAAGAACGGCTGCCAC
GTGGAATTGCTCTTCCTCCGCTACATCTCGGACTGGGACCTAGACCCTGGCCGCTGCTACCGCGTCACCTGGTTCACCTCCTG
GAGCCCCTGCTACGACTGTGCCCGACATGTGGCCGACTTTCTGCGAGGGAACCCCAACCTCAGTCTGAGGATCTTCACCGCGC
GCCTCTACTTCTGTGAGGACCGCAAGGCTGAGCCCGAGGGGCTGCGGCGGCTGCACCGCGCCGGGGTGCAAATAGCCATCATG
ACCTTCAAAGATTATTTTTACTGCTGGAATACTTTTGTAGAAAACCACGAAAGAACTTTCAAAGCCTGGGAAGGGCTGCATGA
AAATTCAGTTCGTCTCTCCAGACAGCTTCGGCGCATCCTTTTGCCCCTGTATGAGGTTGATGACTTACGAGACGCATTTCGTA
CTTTGGGACTTTCCGGACTCAGATCTCGAGCTGATCCAAAAAAGAAGAGAAAGGTAGATCCAAAAAAGAAGAGAAAGGTAGAT
CCAAAAAAGAAGAGAAAGGTA

BsiWI-ZF	ATAGGGGCGTACGGCCACCATGGCTAGCCCCAGAGTGAGAACCGGT
BsrGI-AID	TACTTGTACATTATACCTTTCTCTTCTTTTTTGGATCTACCTTTCTCTTCTTTTTTGGATCT ACCTTTCTCTTCTTTTTTGGATCAGCTCGAGATCTGAGTCCGGAAAGTCCCAAAGTACGAAA TGCGTCTCGTAA
BsrG1-ΔAID	CTTACTTGTACATTATACCTTTCTCTTCTTTTTTGGATCTACCTTTCTCTTCTTTTTTGGAT CTACCTTTCTCTTCTTTTTTGGATCAGCTCGAGATCTGAGTCCGGACAGGGGCAAAAGGATG CGCCGAAG

Supplementary Note 16

ZF$_{GFPIN}$- AID$^{\Delta NES}$ s/ZF$_{GFPIN}$Ns sequence and primers

The SV40 NLS is highlighted in Yellow. ZF_{GFPIN} (ZF_{GFPINL}/ZF_{GFPINR}) modules are in Red, nuclease/deaminase with the linkers is in Blue.

ZF_{GFPINL}- $AID^{\Delta NES}$

ATGGACCTAAGAAAAAGAGGAAGGTGGCGGCCGCTGACTACAAGGATGACGACGATAAATCTAGACCCGGGGAGCGCCCCTT
CCAGTGTCGCATTTGCATGCGGAACTTTTCGCAGGACTCCTCCCTGCGGCGGCATACCCGTACTCATACCGGTGAAAAACCGT
TTCAGTGTCGGATCTGTATGCGAAATTTCTCCCGGCAGGAGCACCTGGTGCGGCATCTACGTACGCACACCGGCGAGAAGCCA
TTCCAATGCCGAATATGCATGCGCAACTTCAGTGACCCCACCTCCCTGAACCGGCATCTGAAGACACATACAGGTGAAAAAGG
ATCCTCTGGTGGTGGACTGGGGTCGACTGACAGCCTCTTGATGAACCGGAGGAAGTTTCTTTACCAATTCAAAAATGTCCGCT
GGGCTAAGGGTCGGCGTGAGACCTACCTGTGCTACGTAGTGAAGAGGCGTGACAGTGCTACATCCTTTTCACTGGACTTTGGT
TATCTTCGCAATAAGAACGGCTGCCACGTGGAATTGCTCTTCCTCCGCTACATCTCGGACTGGGACCTAGACCCTGGCCGCTG
CTACCGCGTCACCTGGTTCACCTCCTGGAGCCCCTGCTACGACTGTGCCCGACATGTGGCCGACTTTCTGCGAGGGAACCCCA
ACCTCAGTCTGAGGATCTTCACCGCGCGCCTCTACTTCTGTGAGGACCGCAAGGCTGAGCCCGAGGGGCTGCGGCGGCTGCAC
CGCGCCGGGGTGCAAATAGCCATCATGACCTTCAAAGATTATTTTTACTGCTGGAATACTTTTGTAGAAAACCACGAAAGAAC
TTTCAAAGCCTGGGAAGGGCTGCATGAAAATTCAGTTCGTCTCTCCAGACAGCTTCGGCGCATCCTTTTGCCCCTGTATGAGG
TTGAT

ZF_{GFPINR}- $AID^{\Delta NES}$

ATGGACCTAAGAAAAAGAGGAAGGTGGCGGCCGCTGACTACAAGGATGACGACGATAAATCTAGACCCGGGGAGCGCCCCTT
CCAGTGTCGCATTTGCATGCGGAACTTTTCGTCCCAGACCCAGCTGGTGCGGCATACCCGTACTCATACCGGTGAAAAACCGT
TTCAGTGTCGGATCTGTATGCGAAATTTCTCCCAGTCCACCACCCTGAAGCGGCATCTACGTACGCACACCGGCGAGAAGCCA
TTCCAATGCCGAATATGCATGCGCAACTTCAGTCAGCGGAACAACCTGGGCCGGCATCTGAAGACACATACAGGTGAAAAAGG
ATCCTCTGGTGGTGGACTGGGGTCGACTGACAGCCTCTTGATGAACCGGAGGAAGTTTCTTTACCAATTCAAAAATGTCCGCT
GGGCTAAGGGTCGGCGTGAGACCTACCTGTGCTACGTAGTGAAGAGGCGTGACAGTGCTACATCCTTTTCACTGGACTTTGGT
TATCTTCGCAATAAGAACGGCTGCCACGTGGAATTGCTCTTCCTCCGCTACATCTCGGACTGGGACCTAGACCCTGGCCGCTG
CTACCGCGTCACCTGGTTCACCTCCTGGAGCCCCTGCTACGACTGTGCCCGACATGTGGCCGACTTTCTGCGAGGGAACCCCA
ACCTCAGTCTGAGGATCTTCACCGCGCGCCTCTACTTCTGTGAGGACCGCAAGGCTGAGCCCGAGGGGCTGCGGCGGCTGCAC
CGCGCCGGGGTGCAAATAGCCATCATGACCTTCAAAGATTATTTTTACTGCTGGAATACTTTTGTAGAAAACCACGAAAGAAC
TTTCAAAGCCTGGGAAGGGCTGCATGAAAATTCAGTTCGTCTCTCCAGACAGCTTCGGCGCATCCTTTTGCCCCTGTATGAGG
TTGAT

ZF_{GFPINL}-N

ATGGACCTAAGAAAAAGAGGAAGGTGGCGGCCGCTGACTACAAGGATGACGACGATAAATCTAGACCCGGGGAGCGCCCCTT
CCAGTGTCGCATTTGCATGCGGAACTTTTCGCAGGACTCCTCCCTGCGGCGGCATACCCGTACTCATACCGGTGAAAAACCGT
TTCAGTGTCGGATCTGTATGCGAAATTTCTCCCGGCAGGAGCACCTGGTGCGGCATCTACGTACGCACACCGGCGAGAAGCCA
TTCCAATGCCGAATATGCATGCGCAACTTCAGTGACCCCACCTCCCTGAACCGGCACCTAAAAACCCACCTGAGGGGATCCCA
ACTAGTCAAAAGTGAACTGGAGGAGAAGAAATCTGAACTTCGTCATAAATTGAAATATGTGCCTCATGAATATATTGAATTAA
TTGAAATTGCCAGAAATTCCACTCAGGATAGAATTCTTGAAATGAAGGTAATGGAATTTTTTATGAAAGTTTATGGATATAGA
GGTAAACATTTGGGTGGATCAAGGAAACCGGACGGAGCAATTTATACTGTCGGATCTCCTATTGATTACGGTGTGATCGTGGA
TACTAAAGCTTATAGCGGAGGTTATAATCTGCCAATTGGCCAAGCAGATGAAATGCAACGATATGTCGAAGAAAATCAAACAC
GAAACAAACATATCAACCCTAATGAATGGTGGAAAGTCTATCCATCTTCTGTAACGGAATTTAAGTTTTTATTTGTGAGTGGT
CACTTTAAAGGAAACTACAAAGCTCAGCTTACACGATTAAATCATATCACTAATTGTAATGGAGCTGTTCTTAGTGTAGAAGA
GCTTTTAATTGGTGGAGAAATGATTAAAGCCGGCACATTAACCTTAGAGGAAGTGAGACGGAAATTTAATAACGGCGAGATAA
ACTTT

ZF_{GFPINR}-N

ATGGACCTAAGAAAAAGAGGAAGGTGGCGGCCGCTGACTACAAGGATGACGACGATAAATCTAGACCCGGGGAGCGCCCCTT
CCAGTGTCGCATTTGCATGCGGAACTTTTCGTCCCAGACCCAGCTGGTGCGGCATACCCGTACTCATACCGGTGAAAAACCGT
TTCAGTGTCGGATCTGTATGCGAAATTTCTCCCAGTCCACCACCCTGAAGCGGCATCTACGTACGCACACCGGCGAGAAGCCA
TTCCAATGCCGAATATGCATGCGCAACTTCAGTCAGCGGAACAACCTGGGCCGGCACCTAAAAACCCACCTGAGGGGATCCCA
ACTAGTCAAAAGTGAACTGGAGGAGAAGAAATCTGAACTTCGTCATAAATTGAAATATGTGCCTCATGAATATATTGAATTAA
TTGAAATTGCCAGAAATTCCACTCAGGATAGAATTCTTGAAATGAAGGTAATGGAATTTTTTATGAAAGTTTATGGATATAGA
GGTAAACATTTGGGTGGATCAAGGAAACCGGACGGAGCAATTTATACTGTCGGATCTCCTATTGATTACGGTGTGATCGTGGA
TACTAAAGCTTATAGCGGAGGTTATAATCTGCCAATTGGCCAAGCAGATGAAATGCAACGATATGTCGAAGAAAATCAAACAC
GAAACAAACATATCAACCCTAATGAATGGTGGAAAGTCTATCCATCTTCTGTAACGGAATTTAAGTTTTTATTTGTGAGTGGT
CACTTTAAAGGAAACTACAAAGCTCAGCTTACACGATTAAATCATATCACTAATTGTAATGGAGCTGTTCTTAGTGTAGAAGA
GCTTTTAATTGGTGGAGAAATGATTAAAGCCGGCACATTAACCTTAGAGGAAGTGAGACGGAAATTTAATAACGGCGAGATAA
ACTTT

BsiWI-ZFL/R	TGGCAAATATTCTGAAATGAGCTGTTGACAATTAATCATCCGGTCCGTATAATCTGTGGAATTGT GAGCGGATAACAATTTCACACAAAGAGGAGAAAGGTACCATGTCGCGGACCCGGCTCCC
BamHI-ZFL/R	AGAGGATCCTTTTTCACCTGTATGTGTCTTCAGATGCCGGCCCAGGTTGTTCCGCTGAC
NheI-ZFL/R	TGGCTAGCACCATGGGACCTAAGAAAAAGAGGAAGGTGGCGGCCGCTGACTACAAGGATGACGAC GATAAATCTAGACCCGGGGAGCGCCCCTTCCAGTGTCG
ApaI-ZFL/R	CTTACCTTCGAAGGGCCCTTAATCAACCTCATAC
